# Synaptic Basis for Contrast-Dependent Shifts in Functional Identity in Mouse V1

**DOI:** 10.1523/ENEURO.0480-18.2019

**Published:** 2019-04-09

**Authors:** Molis Yunzab, Veronica Choi, Hamish Meffin, Shaun L. Cloherty, Nicholas J. Priebe, Michael R. Ibbotson

**Affiliations:** 1 National Vision Research Institute, Australian College of Optometry, Carlton, Victoria 3053, Australia; 2 Department of Optometry and Vision Sciences, University of Melbourne, Parkville, Victoria 3010, Australia; 3 University of Texas Austin, Centre for Learning and Memory, Austin, TX 78712; 4 Department of Physiology, Monash University, Clayton, Victoria 3800, Australia

**Keywords:** complex cell, in vivo whole-cell recording, phase sensitivity, primary visual cortex, visual system

## Abstract

A central transformation that occurs within mammalian visual cortex is the change from linear, polarity-sensitive responses to nonlinear, polarity-insensitive responses. These neurons are classically labelled as either simple or complex, respectively, on the basis of their response linearity (Skottun et al., 1991). While the difference between cell classes is clear when the stimulus strength is high, reducing stimulus strength diminishes the differences between the cell types and causes some complex cells to respond as simple cells (Crowder et al., 2007; van Kleef et al., 2010; Hietanen et al., 2013). To understand the synaptic basis for this shift in behavior, we used *in vivo* whole-cell recordings while systematically shifting stimulus contrast. We find systematic shifts in the degree of complex cell responses in mouse primary visual cortex (V1) at the subthreshold level, demonstrating that synaptic inputs change in concert with the shifts in response linearity and that the change in response linearity is not simply due to the threshold nonlinearity. These shifts are consistent with a visual cortex model in which the recurrent amplification acts as a critical component in the generation of complex cell responses (Chance et al., 1999).

## Significance Statement

The discovery of simple and complex cells in the primary visual cortex (V1) has been fundamental to our understanding of visual processing. While both cell types are orientation selective, simple cells are spatial phase sensitive while complex cells are phase invariant. Extracellular recordings have shown that the responses of complex cells become phase sensitive at lower stimulus contrasts, suggesting more flexibility in processing mechanisms than previously thought. The mechanism by which this flexibility arises is not understood. Using *in vivo* whole-cell recordings, we demonstrated that the flexibility in phase sensitivity is also apparent in the subthreshold responses of mouse V1 cells, suggesting that the effect arises from active cortical recurrent network activity and not from passive spiking threshold mechanisms.

## Introduction

The receptive fields (RFs) of cells in the primary visual cortex (V1) are classified as either simple or complex based on their spatial organization (Hubel and Wiesel, 1962; Henry, 1977). Simple cell RFs have segregated subfields that respond to either brightness increments (ON) or decrements (OFF); complex cells do not have clearly segregated ON and OFF subfields (Hubel and Wiesel, 1962; Gilbert, 1977; Henry, 1977; Hammond and Ahmed, 1985; Spitzer and Hochstein, 1988; Mechler and Ringach, 2002; Priebe et al., 2004; Hietanen et al., 2013). The Hubel and Wiesel hierarchical model proposed that convergent synaptic inputs are responsible for these transformations in two stages (Hubel and Wiesel, 1962): thalamic relay cells, displaced along an oriented axis, converge on simple cells, generating orientation selectivity, then simple cells converge on complex cells to provide polarity invariance.

The distinction between simple and complex cells is related to neuronal laminar position and synaptic connectivity in some mammals (Ringach et al., 2002; Martinez et al., 2005; Williams and Shapley, 2007). Simple cells are found more often in cortical layers that receive thalamocortical connections, while complex cells are found in layers with dense recurrent cortical connectivity. The differences between simple and complex cell RFs may reflect a general process in which cortical circuits generalize selectivity by amplifying inputs. While cortical amplification has previously been hypothesized to increase selectivity (Ben-Yishai et al., 1995; Douglas et al., 1995; Somers et al., 1995), it is also possible for it to generalize selectivity by integrating inputs with distinct RFs. We examined whether simple and complex cell responses in V1 exhibited signatures of this amplification.

One quantitative method to distinguish simple and complex cells depends on the relative modulation of responses to drifting sinusoidal gratings (Skottun et al., 1991). When stimulated with high-contrast drifting gratings, simple cell responses modulate as the grating moves across the distinct ON and OFF subfields. In contrast, complex cells respond to all phases of the drifting gratings. Studies have demonstrated that the ratio (F_1_/F_0_) of the modulated spiking component (F_1_) to the unmodulated component (F_0_) forms a bimodal distribution, suggesting two classes of V1 neurons (Maffei and Fiorenti, 1973; Movshon et al., 1978; De Valois et al., 1982; Skottun et al., 1991). While this difference between cell classes is clear when the stimulus strength is high, reducing stimulus strength diminishes the differences between the cell types. In particular, low contrast gratings evoke modulated responses in many complex cells (cat: Crowder et al., 2007; van Kleef et al., 2010; monkey: Henry and Hawken, 2013; Cloherty and Ibbotson, 2015; Meffin et al., 2015).

The mechanism underlying this change in spiking modulation ratio is not understood but there are two candidate models. The first model suggests that modulations in response to low contrast stimuli emerge due to the “iceberg” effect in which not all synaptic responses are converted into spikes (Carandini and Ferster, 2000; Mechler and Ringach, 2002; Priebe et al., 2004). In this model the subthreshold synaptic modulation ratio (V_1_/V_0_) should not depend on contrast. Alternatively, there may be a shift in the synaptic inputs to V1 neurons in which the V_1_/V_0_ ratio increases as the contrast decreases. A cortical model in which the amplification acts to integrate inputs with distinct spatial preferences predicts this specific change in synaptic input.

To distinguish these possibilities, we performed *in vivo* whole-cell recordings in mouse V1. Both the V_1_/V_0_ and the F_1_/F_0_ ratios increased as contrast was reduced indicating that a change in synaptic drive is the more likely explanation for the altered modulation responses of complex cells. We have demonstrated that the circuitry leading to spatial phase invariant responses in visual cortex depends on the strength of visual drive. This observation is consistent with a scheme of complex cell generation in which the recurrent inputs in the visual cortex act as amplifiers, generating linear or nonlinear responses when input gain is low or high, respectively (Chance et al., 1999).

## Materials and Methods

### Electrophysiology

Recordings were made from anaesthetized C57BL/6 mice of both sexes aged five to twelve weeks. All experiments were performed according to the National Health and Medical Research Council’s Australian Code of Practice for the Care and Use of Animals for Scientific Purposes. All experimental procedures were approved by Animal Ethics Committees of the University of Melbourne, or by the Institutional Animal Care and Use Committee of the University of Texas at Austin. Mice were anesthetized with intraperitoneal injections of chloroprothixene (10 mg/kg) followed by urethane (1 g/kg). Animals also received an injection of dexamethasone (2 mg/kg) to reduce brain edema. The level of anesthesia was monitored using the electrocardiogram (ECG) and repeated toe-pinches throughout the experiment. Body temperature was monitored and maintained at 37°C using an auto-regulating heating blanket. A tracheotomy was performed to ensure a clear airway. A craniotomy ∼1 × 2.5 mm was performed over V1 in one hemisphere and the dura mater retracted.

Intracellular responses were obtained in mice via blind recordings with a whole-cell configuration *in vivo* as previously described (Ferster and Jagadeesh, 1992; Margrie et al., 2002; Priebe et al., 2004; Tan et al., 2011). Patch pipettes with tip resistances of 8–10 MOhm were pulled from borosilicate glass capillaries (1.2 mm outer diameter, 0.7 mm inner diameter; KG-33, King Precision Glass). A silver chloride coated silver wire was inserted into the pipette, which was filled with 135 mM K-gluconate, 4 mM NaCl, 0.5 mM EGTA, 2 mM MgATP, 10 mM phosphocreatine disodium, and 10 mM HEPES; pH adjusted to 7.3 with KOH (Sigma-Aldrich). A silver–silver chloride wire was inserted as a reference electrode into muscles near the base of the skull. The craniotomy as well as the reference electrode was covered with 4% agarose in normal saline to keep the cortex moist and to reduce changes in the surrounding fluid and concomitant changes in associated junction potentials. An Axoclamp 2B patch-clamp amplifier was used in current clamp to record from neurons 150–600 μm below the surface of the cortex. The voltage was digitized and recorded with custom software (Labview, National Instruments), which also sent instructions to a separate stimulus-generation computer.

### Visual stimuli

Visual stimuli were generated using the Psychophysics toolbox for MATLAB (The MathWorks Inc.) and were presented on a calibrated CRT monitor (Sony GDM-F520, 100 Hz non-interlaced refresh rate, 1280 × 1024 pixels, 25 cd/m^2^ mean luminance). The viewing distance for all recordings was 30 cm. For each recorded cell we measured its orientation, spatial frequency (SF), and temporal frequency (TF) preferences, as well as its RF location and size using drifting sinusoidal gratings. For example, to determine orientation preference, sinusoidal gratings were presented at eight different orientations (0°, 22.5°, 45°, 67.5°, 90°, 112.5°, 135°, 157.5°). After 0.5 s of presentation of each orientation, gratings moving in the opposite direction were presented and followed with 0.5 s of gray screen (at the mean luminance of the prior grating). The optimal tuning parameters were determined online and then applied to the experimental stimuli. The contrast of the grating was defined as: Michelson contrast = [(Lum_max_ – Lum_min_)/Lum_max_ + Lum_min_)] × 100, where Lum_max_ and Lum_min_ are the maximum and minimum luminance of the grating.

Two types of experimental stimuli were used: drifting sinusoidal gratings and sinusoidally modulated contrast-reversing gratings. Stimuli were presented at the optimal TF, SF, and orientation of the recorded cell in a circular aperture the size of its excitatory RF. Drifting gratings with contrast levels ranging between 4% and 100% were presented in pseudorandom order interleaved with 1-s presentations of a blank (mean luminance) screen. Each grating was presented for 3 s with the first and last 0.5 s stationary, and drifting for the 2 s in between. Trials were repeated as many times as the stability of the recording would allow. Contrast-reversing gratings were presented at 8 different spatial phases (0°, 45°, 90°, 135°, 180°, 225°, 270°, 315°). Depending on the recording stability, various combinations of contrast between 8% and 100% were tested. Each stimulus presentation consisted of a grating presented for 0.5 s with a steady contrast, 2 s presented with sinusoidally modulated contrast, and another 0.5 s with steady contrast.

### Data analysis

The resting membrane potential (V_rest_) of a patched cell, measured as the responses to a blank screen (0% contrast), ranged from –40 to –80 mV. To examine the subthreshold membrane potential modulation, spikes were removed from the raw records before analysis using a 5 ms median filter (Jagadeesh et al., 1997). The modulation ratios for membrane potential (V_1_/V_0_) and spiking rate (F_1_/F_0_) to drifting gratings were calculated as previously described in Priebe et al. (2004). For contrast-reversing gratings, the modulation ratios for membrane potential and spiking rate were calculated as V_2_/V_1_ and F_2_/F_1_, respectively. Cycle-averaged responses were measured by aligning each response cycle, excluding the first cycle. The mean and standard error of the membrane potential and spiking rate were calculated at each time point of the cycle-averaged response. As in Priebe et al. (2004), the mean membrane potential (V_0_) and spiking rate (F_0_) are based on the differences between the responses during a stimulus and the spontaneous responses during a blank screen of the same time. Fourier coefficients at the fundamental frequency of the stimulus grating (V_1_ for membrane potential, F_1_ for spiking rate) and at twice the stimulus input (V_2_ for membrane potential, F_2_ for spiking rate) for each cycle-averaged response were extracted using the FFT function in MATLAB (The MathWorks Inc.). A perfect half-wave rectified spiking rate response is expected to have an F_1_/F_0_ ratio of 1.57. We found two cells with F_1_/F_0_ ratios above 1.57 at high stimulus contrasts, but this was due to low F_0_ values created by subtracting the high spontaneous spiking rate from the evoked spiking rate. All cells showed significant increases in mean spiking rate (F_0_) relative to the spontaneous spiking rate (*p* < 0.05, one-sided *t* tests). One cell with a modulation ratio (F_1_/F_0_) of 2.67 had a relatively high spontaneous spiking rate (6.9 spikes/s) compared to its evoked spiking rate (9.6 spikes/s). The other cell with F_1_/F_0_ of 1.98 showed a relatively high spontaneous spiking rate (1.2 spikes/s) compared to its evoked spiking rate (6.5 spikes/s). Both cells showed significant increases in evoked F_1_ amplitude with a clear response to the drifting grating stimulus (*p* = 0.0002 and *p* < 0.0001, one-sided *t* tests). Error bars were generated by projecting the cycle-by-cycle estimate of modulation amplitude and phase onto the mean phase and amplitude vector in complex space.

### Model

Each neuron in the network model receives feedforward and recurrent input and is based on the rate model developed by Chance et al. (1999). The activity of neuron *i*, is modeled using a simple rate model equation that includes a threshold nonlinearity:

(1) $$\tau_{v}\frac{dv_{i}}{\mathrm{dt}}=I_{i}^{\mathrm{ff}}+I_{i}^{\mathrm{rec}}-v_{i}$$

(2) $$r_{i}={\left\lfloor v_{i}-v_{\mathrm{thresh}}\right\rfloor }_{+}$$

where $I_{i}^{\mathrm{ff}}$ and $I_{i}^{\mathrm{rec}}$ represent the feedforward and recurrent inputs. We use a time constant, $\tau_{r}$ of 20 ms and a positive voltage threshold ($v_{\mathrm{thresh}}$). The feedforward input is equal to a half-wave rectified sinusoidal modulation, where each network neuron has a random preferred spatial phase ($\phi_{i}$).

(3) $$I_{i}^{\mathrm{ff}}=\rho \sin\left(\frac{2^{*}\pi t}{500}+\phi_{i}\right)$$

The recurrent input to model neuron *i* is given by:

(4) $$I_{i}^{\mathrm{rec}}=\frac{\left(1-\rho_{i}\right)}{N}\sum r_{i}$$

The degree of recurrent and feedforward input is set by the value of $\rho_{i}$, which was randomly set between 0 and 1, with zero reflecting all recurrent input and one reflecting all feedforward input.

## Results

**Figure 1.**
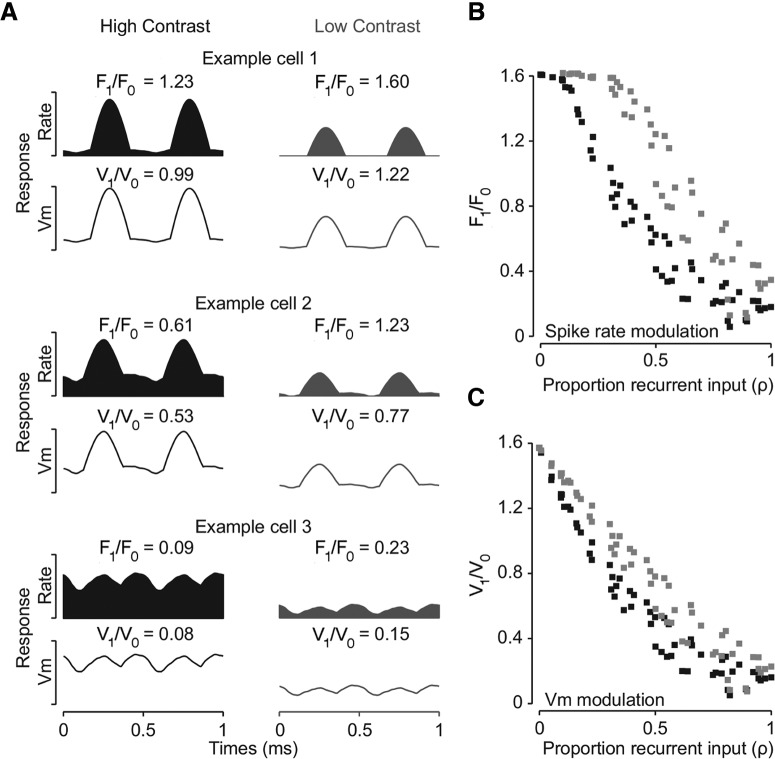
Model for contrast-dependent transformation between simple and complex cells in mammalian V1 neurons. ***A***, Example of a model cell with a modulation ratio (F_1_/F_0_) that is greater than 1 (example cell 1, top), a model cell with F_1_/F_0_ between 0.5 and 1 (example cell 2, middle), and a cell with F_1_/F_0_ below 1 (example cell 3, bottom) at high contrasts. The top row represents the spiking rate of the cell and the bottom row is the underlying membrane potential of the cell. The black trace to the left shows responses of the cell to high contrasts and the light grey trace to the right shows responses to low contrasts. ***B***, Black dots represent spiking rate at high contrast and grey dots represent spiking rate at low contrast. As the proportion of recurrent input increases (high contrast) the modulation ratio derived from the spiking rate (F_1_/F_0_) decreases. ***C***, same as ***B*** but for membrane potential.

We first explored a model of the transformation between simple and complex cells to guide our experiments based on the architecture from Chance et al. (1999). They used a rate model to demonstrate that the degree of simple cell and complex cell behavior is related to the amount of recurrent circuitry in the network. We implemented their model with two changes. First, each neuron received a random degree of feedforward input ($\rho_{i}$) so that we would observe both simple and complex cells within the same network. Second, we included a non-zero threshold to model the threshold nonlinearity between the input and output. Both simple and complex cells emerge from this network model, as seen in the modulation ratio to a drifting grating (Fig. 1*A*). Simple cells respond at one phase of the stimulus and have a modulation ratio that is >1 (Fig. 1*A*, top row). Network complex cells exhibit a response that varies little with the drifting grating and are characterized by a modulation ratio <1 (Fig. 1*A*, bottom row). Importantly in this network simulation we can view neurons that exhibit combinations of linear and nonlinear components (Fig 1*A*, middle row) and therefore have a modulation ratio between 0.5 and 1.

As the emergence of complex cells in this model depends on the degree of recurrent amplification, we hypothesized that reducing the input strength would impact the modulation ratios of network neurons, and thus the degree of generalization across spatial phase. Indeed, we find that reducing input strength, or visual contrast, leads to systematic increases in the modulation ratios of network neurons (Fig. 1*B*). The modulation ratios of model neurons shift to higher values as the input strength is reduced, even switching neurons that would be classified as complex for high contrast to being simple at low contrast (Fig. 1*A*, middle row). Contrast-dependent changes in the modulation ratio could therefore reflect the amplification structure of the model visual cortex. There are two components that contribute to the increase in modulation ratio with contrast. First, the observed increase in the network modulation ratio with contrast depends on the voltage threshold (V_thresh_). If the voltage threshold is set to 0 then no change in modulation ratio occurs with changes in contrast (data not shown). Second, there are systematic increases in the synaptic modulation ratio (Fig. 1*C*) as contrast is reduced. This simple model demonstrates how the cortical circuitry, acting as an amplifier, could generate spatially invariant responses and demonstrates that the degree of the spatial invariance depends on the input strength.

**Figure 2.**
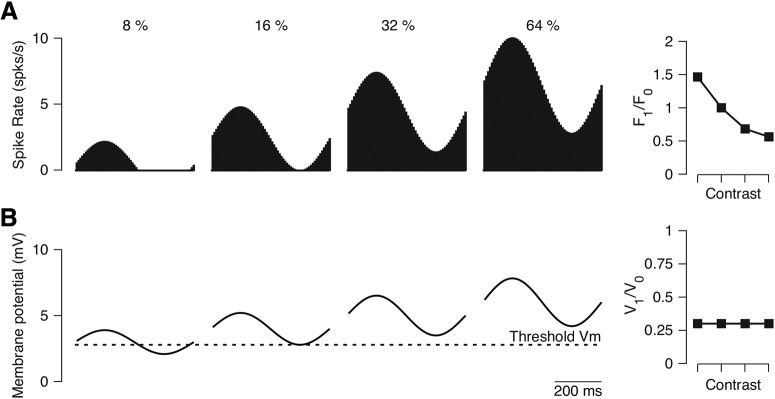
Threshold nonlinearity model (“iceberg” model) for a contrast-dependent transformation between simple and complex cells in mammalian V1 neurons. ***A***, Spiking responses of a model neuron relative to contrast. As contrast increases, the spiking rate of the model neuron increases (right). The modulation ratio (F_1_/F_0_) decreases with increasing contrast due to relatively large increases in the mean spiking rate (F_0_) compared to the F1 component. ***B***, Membrane potential responses of the model neuron relative to contrast. As the contrast increases, the amplitude of the membrane potential increases. The matched increases in the V_0_ and V_1_ components of the responses generate a steady modulation ratio (V_1_/V_0_) at all contrasts.

There are other possible models that could explain the shift in modulation ratio due to changes in contrast. One alternative possibility is an iceberg effect where not all synaptic responses are converted into spiking activities (Carandini and Ferster, 2000; Mechler and Ringach, 2002; Priebe et al., 2004). For a high contrast stimulus, the synaptic input is sufficient to evoke spiking responses at all phases (Hietanen et al., 2013), whereas for a low contrast stimulus, the synaptic input falls below threshold and is only sufficient to evoke spiking responses for a subset of phases (Fig. 2*A*). In this model, the modulation ratio of the synaptic input (V_1_/V_0_: the modulation ratio of the membrane potential) does not vary (Fig. 2*B*); instead the change in the spiking modulation ratio is due to the threshold nonlinearity. This explanation for the observed changes in the spike modulation ratio with contrast proposes that the underlying membrane potential modulation ratio is fixed and the changes observed at the level of spiking emerge from the threshold nonlinearity.

To examine whether signatures of these models exist in V1 neuron responses we measured the degree to which the modulation ratio of the synaptic input varies with contrast. Previous experimental reports have demonstrated that the spiking modulation ratio of V1 neurons is contrast dependent, which matches the pattern shown in the model, i.e., responses become more simple-like as contrast declines. However, these records do not differentiate between synaptic changes from the network and changes that may exclusively emerge from threshold nonlinearity. To determine whether the change in the spiking modulation ratio is due to threshold or synaptic mechanisms, we recorded intracellularly from V1, giving us access to both the underlying membrane potential as well as the spiking rate in response to gratings. We recorded from 20 cells with drifting gratings and 21 cells with contrast reversing gratings in 20 urethane-anaesthetized mice.

### Responses to drifting gratings

**Figure 3.**
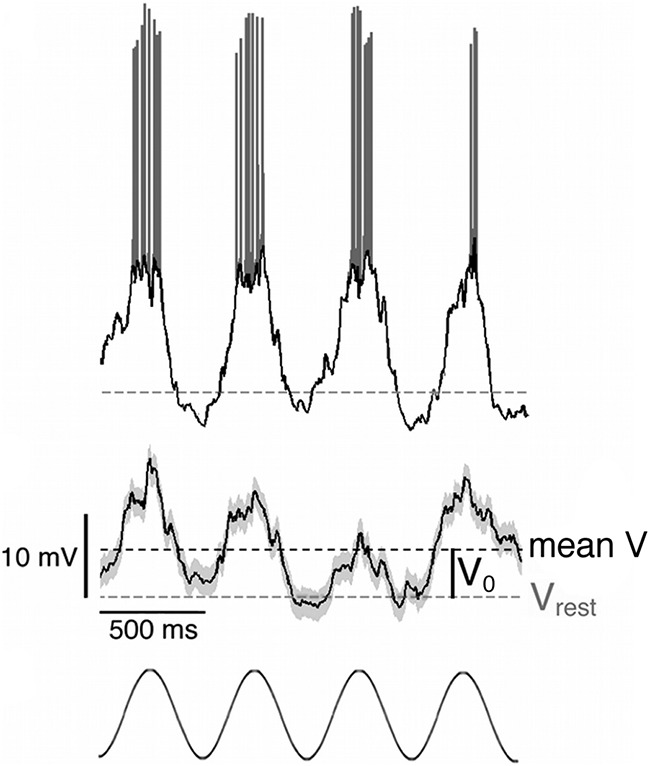
Intracellular responses of a mouse V1 simple cell to a drifting sinusoidal grating. Top panel: raw response to a sinusoidal grating with 100% contrast moving at 2 Hz at the cell's preferred orientation. The black trace represents the subthreshold membrane potential; spikes are shown in grey. The broken line indicates the resting membrane potential of the cell: −48.5 mV. Middle panel: trial-averaged (5 repeats) subthreshold voltage trace for the same cell, including the data in the top panel. Spikes are removed from voltage traces prior to averaging by calculating the derivative of the membrane potential and excluding rapid voltage excursions based on a derivative threshold. The grey shading shows the standard error of the mean. The mean membrane potential (mean V) and resting membrane potential (V_rest_) for the trial-averaged subthreshold responses are shown respectively as dark grey and light grey broken lines. The voltage difference between the mean and resting membrane potential is defined as V_0_. Bottom panel: A visual representation of 4 cycles of the sinusoidal grating stimulus moving at 2 Hz (2 s in total).

Based on responses to drifting sinusoidal gratings, mouse V1 neurons show the same separation into simple and complex cells as cats and primates (Niell and Stryker, 2008). We classified cells as simple by the large modulation of spiking rate (F_1_/F_0_ > 1) to a drifting grating stimulus. The underlying membrane potential of these neurons also exhibited large modulations when stimulated at the preferred orientation, SF and TF (100% contrast; Fig. 3). Membrane potential fluctuations were separated from spiking rate by identifying the spike times and removing them from the membrane potential traces using a median filter (see Materials and Methods). Both the raw response and the trial-averaged membrane potential for the simple cell in Figure 3 are highly modulated at the input frequency and phase-locked to the sinusoidal grating stimulus (Fig. 3, bottom).

**Figure 4.**
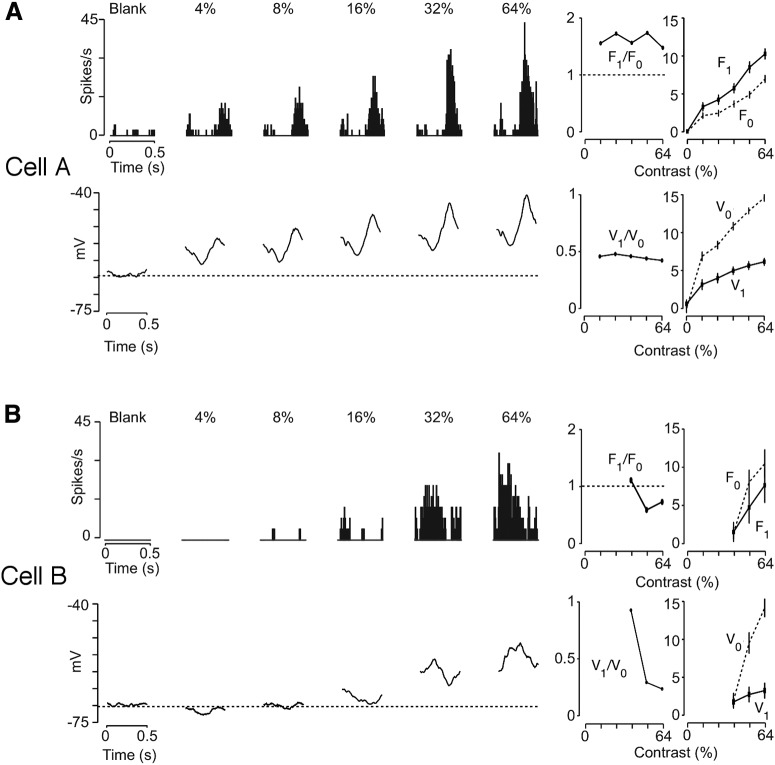
The responses of two example mouse V1 cells (Cell A and Cell B) to drifting gratings (***A*** & ***B***). Left panel: cycle-averaged spiking rate (top panel) and membrane potential (bottom panel) responses to drifting sinusoidal gratings at different stimulus contrasts. From left to right, responses to a blank screen and five stimulus contrasts (blank, 4%, 8%, 16%, 32% and 64%) are shown for each cell. The broken lines represent spontaneous spiking rate and resting membrane potential (V_rest_) for spiking rate (top panel) and membrane potential (bottom panel) responses, respectively. Right panel: The spiking modulation ratios (F_1_/F_0_, top panel) and the membrane potential modulation ratios (V_1_/V_0_, bottom panel) plotted as functions of contrast. The unity line, where F_1_/F_0_ = 1, is indicated as a broken line in the top panel. F_0_ and F_1_ values (top panel) and V_0_ and V_1_ values (bottom panel) are shown as functions of contrast.

**Figure 5.**
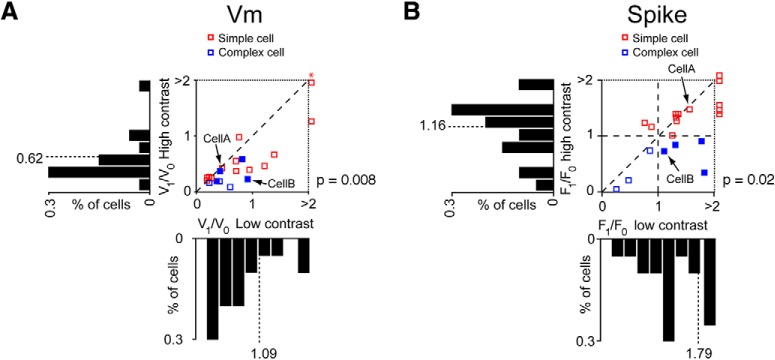
Population results of mouse V_1_ cells recorded with drifting gratings (*n* = 20). ***A***: Scatter plots of membrane potential modulation ratios (V_1_/V_0_) and ***B***: spiking modulation ratios (F_1_/F_0_) at low (16%) and high (64%) contrasts. The diagonal broken lines indicate the unity lines, where the modulation ratios are the same at high and low contrasts. Compared to high contrasts, the modulation ratios showed significant increases at low contrasts in both membrane potential (one-sided *t*-test, *p* = 0.008) and spiking rate (one-sided t-test, *p* = 0.02). Red symbols represent cells that have F_1_/F_0_ > 1 (simple cells) at high contrast. Blue symbols represent cells that have F_1_/F_0_ < 1 (complex cells) at high contrast. In ***B***, the horizontal and vertical broken lines indicate F_1_/F_0_ = 1. Data from the example cells in Figure 4 are marked as Cell A and Cell B on both ***A*** and ***B***. Histograms of distributions of modulation ratios at high and low contrasts are plotted on the same scale next to the corresponding scatter plot axis. The broken lines indicate the means of the distributions. (***A***: * V_1_/V_0_ at low contrast: 4.55, V_1_/V_0_ at high contrast: 7.22).

Previous work has demonstrated that the classification of simple cells does not vary with contrast in the cat and primate (cat: Crowder et al., 2007; van Kleef et al., 2010; monkey: Henry and Hawken, 2013; Cloherty and Ibbotson, 2015; Meffin et al., 2015). We first examined whether this is also true in mouse V1 by measuring the changes in the modulation ratios for spiking rate (F_1_/F_0_) and membrane potential (V_1_/V_0_) of individual simple cells with contrast (Fig. 4*A*). For simple cells, V_1_/V_0_ did vary with contrast but the F_1_/F_0_ was consistently higher than unity, indicating that simple cell classification does not depend on contrast (Fig. 4*A*). Across our sample population we found that the subthreshold modulation ratio (V_1_/V_0_) of simple cells often increased with decreasing contrast but this change was not statistically significant (*n* = 13, *p* > 0.05, one-sided *t* test; Fig. 5*A*, red symbols). This result is consistent with results from an earlier study, in which simple cells in cat V_1_ showed increased V_0_ and V_1_ as contrasts decreased (Carandini and Ferster, 1997). Despite those subthreshold changes, however, the F_1_/F_0_ ratio was consistently above unity for simple cells at low and high contrasts (Fig. 5*B*). Therefore, the simple cell population remains highly phase sensitive at both the membrane potential and spiking output levels for all contrasts.

We next examined how contrast alters the modulation ratio of complex cells in mouse V1. As found in other mammals, complex cells modulate more at low contrasts than high contrasts (cat: Crowder et al., 2007; van Kleef et al., 2010; monkey: Henry and Hawken, 2013; Cloherty and Ibbotson, 2015; Meffin et al., 2015). We found a range of contrast-dependent shifts in the modulation ratios, which demonstrate that synaptic mechanisms are involved in this process. For some complex cells modulations in response amplitude are clearly evoked across all contrasts at the level of both the membrane potential and spiking rate (Fig. 4*B*). Measures of the modulation ratios of the membrane potentials systematically increase as contrast decreases. This shift is matched by a commensurate increase in the F_1_/F_0_ ratios. For both spiking rate and membrane potential responses, the mean responses (F_0_ and V_0_) dominate the modulation amplitudes (F_1_ and V_1_) at high contrasts (Fig. 4*B*). As the contrast decreases, however, the differences between these two parameters declines and results in increased modulation ratios. This trend is especially prominent in the membrane potential responses in which V_1_ remains largely unchanged compared to V_0_. When considering the complex cell and the simple cell examples together, it is noticeable that similar membrane potential characteristics in the two example cells (Fig. 4*A*,*B*) are observed at higher contrasts (32% and 64%). Both cells show substantial modulations of the fundamental frequencies of the input (V_1_) that are well above the resting membrane potential, which result in the V_0_ component being larger than the V_1_ component. However, the spiking responses show different response characteristics in the two cells: cell A has a larger F_1_ component at high contrasts, whereas cell B has a larger F_0_ component (Fig. 4*A*,*B*). As a result, cell A has an F_1_/F_0_ > 1 and is therefore classified as a simple cell, whereas cell B is classified as a complex cell because it has an F_1_/F_0_ < 1. These observations suggest that the dichotomy between simple and complex cells based on spiking modulation ratios with high stimulus strengths does not directly translate to corresponding distinctions in the membrane potential responses. The differences in the F_1_/F_0_ ratios in the two cells are likely the result of non-linear threshold transformations from the membrane potentials to the spiking outputs (Priebe et al., 2004).

At the population level, changes in the F_1_/F_0_ (spiking rate) and V_1_/V_0_ (membrane potential) ratios of complex cells at high and low contrasts have characteristics similar to the responses of the synaptic model. The scatter plots of both F_1_/F_0_ and V_1_/V_0_ show significant increases at low contrast compared to high contrast (Fig. 5). The distribution of V_1_/V_0_ ratios presented as a histogram reveal a significant shift toward higher values at low contrasts compared to high contrasts (*n* = 20, *p* = 0.008, one-sided *t* test; Fig. 5*A*, blue symbols). The population spiking responses also show significant increases in F_1_/F_0_ ratios at low contrasts (*n* = 20, *p* = 0.02, one-sided *t* test; Fig. 5*B*).

### Responses to contrast reversing gratings

**Figure 6.**
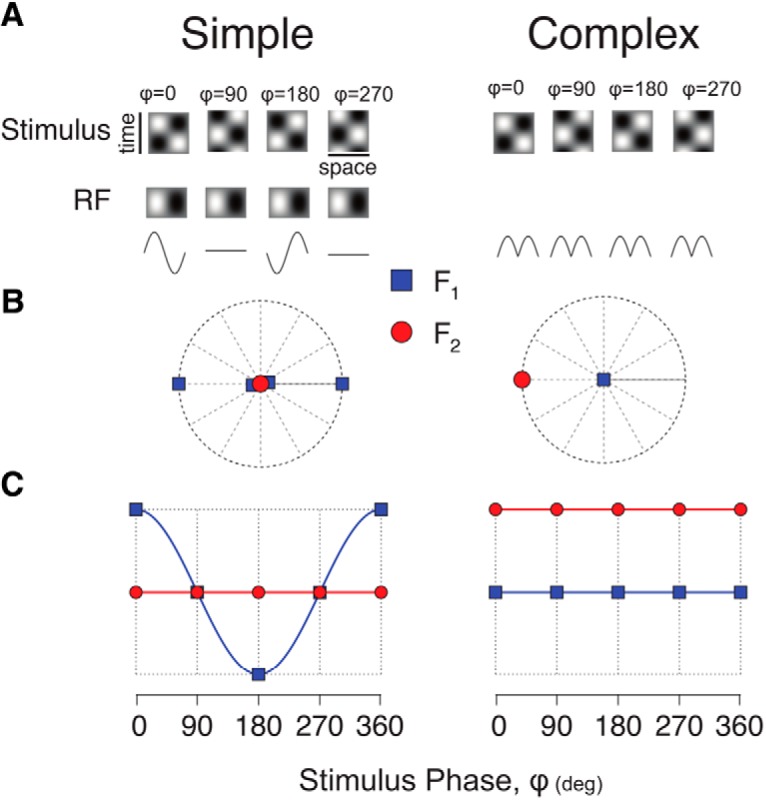
Model responses of a simple and a complex cell to contrast-reversing gratings. ***A***, Simple cell models (left) and complex cell models (right) have distinct responses to contrast reversing gratings. Four different spatial phases of the contrast reversing grating are shown in the top row and the receptive fields of the simple cell are shown below. The receptive field for the complex cell is not shown but reflects the quadrature pairs of simple cell receptive fields (Adelson and Bergen, 1985). Model responses to the contrast reversing grating are shown in the third row. ***B***, Complex plane representation of the responses of the simple cell (left) and complex cell (right) at the first (F_1_, blue square) and second (F_2_, red circle) Fourier frequency. ***C***, Amplitudes of the F_1_ and F_2_ frequencies projected onto the principle axis (see results).

An alternative method to quantify the nonlinearities of cortical neurons is to examine the modulated responses to contrast-reversing gratings (Hawken and Parker, 1987). An ideal simple cell should modulate at the TF of the contrast reversal (F_1_), and the timing of its response should depend on the spatial phase of the grating (Fig. 6, left). An ideal complex cell should modulate at twice the TF of the contrast reversal (F_2_), and the timing of its response should not depend on the spatial phase of the grating (Fig. 6, right). One can then distinguish simple and complex cells by considering the first and second Fourier components in the complex plane (Fig. 6*B*). Simple cells should have large F_1_ components that lie along an axis in the complex plane. For the example simple cell, that axis is along the abscissa. In contrast, the example complex cell has small F_1_ components, but a large F_2_ component for which the response does not change with the stimulus phase. To extract a metric that describes the relative F_1_ and F_2_ modulations of the responses, we computed the amplitude of the projection of the F_1_ values onto their principle axis in the complex plane, and compared that to the vector average F_2_ value in the complex plane. Doing so enforces the expectation that the timing of the F_2_ component should be invariant to spatial phase. The resulting contrast reversing modulation index (F_2_/F_1_) is large for complex cells and small for simple cells.

**Figure 7.**
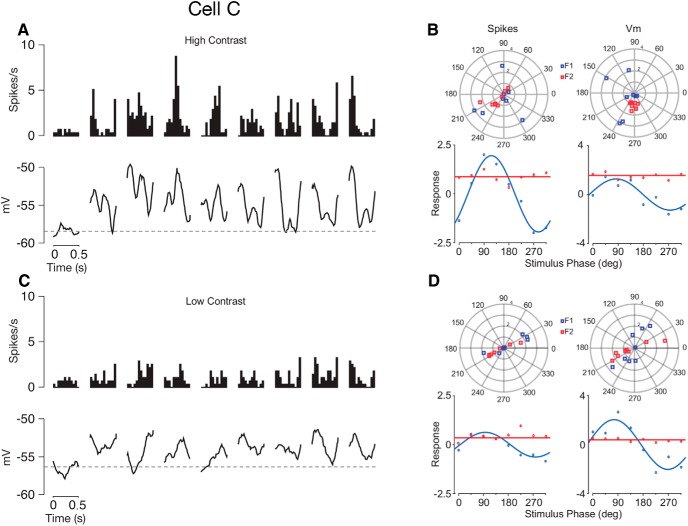
Responses of an example mouse V1 cell (Cell C) to contrast-reversing gratings. ***A***, Cycle-averaged spiking rate (top) and membrane potential (bottom) to contrast-reversing gratings at high (100%) stimulus contrasts for eight different spatial phases. From left to right, responses to a blank screen and spatial phases 0°, 45°, 90°, 135°, 180°, 225°, 270° and 315° are shown. ***B***, F_1_ (blue) and F_2_ (red) values calculated from spiking rate (left panels) and membrane potential (right panels) are plotted separately as functions of spatial phases for high (100%) stimulus contrasts. ***C***, Similar to ***A***, but panels show responses at low (22%) stimulus contrast. ***D***, Similar to ***B*** but at low (22%) stimulus contrast.

To quantify how much V1 neurons shift to more simple-like behavior as contrast is lowered, we presented contrast-reversing gratings at eight different spatial phases and extracted the phase and amplitude of the Fourier components at the TF of the reversing gratings and at twice the TF of the reversing gratings (Fig. 7). These measurements were made both for the spiking rate of the neurons and their underlying membrane potentials.

As with drifting gratings we found that reductions in contrast caused systematic changes in membrane potential modulations that reflected a shift toward more simple-like behavior in complex cells. At high contrast these complex cells are characterized by frequency doubled responses in both membrane potential and spiking rate (Fig. 7*A*). When contrast was lowered, however, the amplitude of the frequency doubled responses declined relative to the modulation at the TF of the reversing grating. Note that not only do modulations emerge at low contrasts, but the timing of the modulations systematically shifts with the spatial phases of the gratings, as predicted by an ideal simple cell (Fig. 6).

**Figure 8.**
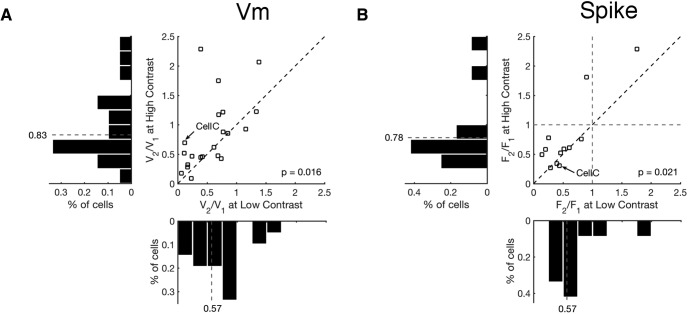
Population results of mouse V1 cells recorded with contrast-reversing gratings. ***A***, ***B***, Scatter plots of membrane potential modulation ratios (V_2_/V_1_) (***A***) and spiking modulation ratios (F_2_/F_1_) (***B***) at low (22% or 32%) and high (100%) contrasts. The diagonal broken lines indicate the unity lines, where the modulation ratio is the same at high and low contrasts. In ***B***, the horizontal and vertical broken lines indicate F_2_/F_1_ = 1. Compared to high contrasts, the modulation ratios showed significant increases at low contrasts in both membrane potential responses (*n* = 21, one-sided *t*-test, *p* = 0.016) and spiking rate (*n* = 12; one-sided *t*-test, *p* = 0.021). Data from the example cell in Figure 7 is marked as Cell C on both ***A*** and ***B***. Histograms of distributions of modulation ratios at high and low contrasts are plotted on the same scale next to the corresponding scatter plot axis. The broken lines indicate the means of the distributions.

As shown in the membrane potential and spiking rate traces, the projected F_2_ and F_1_ modulations vary across stimulus phase (Fig. 7*B*). To quantify the changes outlined above for each cell, we estimated individual F_1_ and F_2_ (spikes) and V_1_ and V_2_ (membrane potentials) values across all spatial phases for each stimulus contrast tested. For F_2_ and V_2_, we simply averaged across all stimulus spatial phases since these values were spatial phase invariant (Figs. 6*C*, 7*B*, red lines). However, averaging across all stimulus spatial phases does not work for F_1_ and V_1_ because they are spatial phase dependent (Figs. 6*C*, 7*B*, blue lines). At high contrasts the amplitudes of the F_2_ and V_2_ modulations do not modulate with spatial phase, while the F_1_ and V_1_ components clearly modulate. When contrast is lowered, the F_2_ and V_2_ modulation amplitudes decline more rapidly relative to the F_1_ and V_1_ components, respectively. These changes cause an overall decline in the membrane potential modulation ratio (V_2_/V_1_) from 0.7 at high contrast to 0.12 at low contrast (Fig. 8*A*, arrow). As small modulation ratios are associated with simple cells and larger ones with complex cells, this is an example in which contrast shifts the behavior of the neuron toward more simple-like responses.

To quantify how contrast altered the behavior of our complex cell population we estimated V_2_/V_1_ and F_2_/F_1_ modulation indices across our sample population (membrane potential: *n* = 21, spikes: *n* = 12; Fig. 8). We found that the membrane potential modulation ratio systematically declined with contrast, changing from a mean value of 0.83–0.57 (*p* = 0.016, one-sided *t* test). There was a similar, but more modest, change in the modulation ratios obtained from spiking rate across our sample population (*p* = 0.021, one-sided *t* test). Therefore, as with drifting gratings, neurons in mouse visual cortex shift toward more simple-like responses as contrast is lowered.

## Discussion

Contrast-dependent phase sensitivity has been documented in a subpopulation of neurons in V1 of cat (Crowder et al., 2007; van Kleef et al., 2010; Hietanen et al., 2013) and primate (Henry and Hawken, 2013; Cloherty and Ibbotson, 2015). The current study demonstrates that contrast-dependent phase sensitivity is also present in the V1 of mouse. Cortical visual processing in mice has been studied extensively in the past decade (Niell and Stryker, 2008; Huberman and Niell, 2011; Tan et al., 2011). The abundant opportunities for genetic manipulation have made mouse visual cortex a useful model in addition to carnivores and primates for studying RF properties (Wang et al., 2006; Liu et al., 2009; Zariwala et al., 2011). All previous literature showing contrast-dependent changes in response linearity in cats and monkeys has been quantified using modulation ratios calculated from extracellular responses to drifting sinusoidal gratings (Crowder et al., 2007; van Kleef et al., 2010; Henry and Hawken, 2013; Hietanen et al., 2013; Cloherty and Ibbotson, 2015) or contrast-reversing gratings (Meffin et al., 2015).

We used whole-cell recordings to shed light on how complex cells emerge in V1. A simple model to describe this process is that recurrent cortical connectivity between neurons with distinct spatial selectivity generates the spatial-invariant responses that characterize complex cells. Two models have been proposed to describe this shift, one from Hubel and Wiesel in which simple cells receive inputs from the dorsal lateral geniculate nucleus (dLGN) in the thalamus and converge onto complex cells in one step to generate spatial invariance (Hubel and Wiesel, 1962). Alternatively the generation of spatial invariance may require many steps, which reflect an increase in the proportion of cortical circuitry that neurons receive (Chance et al., 1999). One way to distinguish these possibilities is to observe how input strength alters the emergence of complex cells. Mouse dLGN neurons show mostly linear contrast sensitivity, the responses of individual dLGN cells increase with increasing contrasts (Grubb and Thompson, 2003; Tang et al., 2016). Lien and Scanziani (2013) have shown that recurrent cortical excitation to simple cells in mouse V1 is phase-sensitive and matches the dLGN inputs. However, it is unclear whether this is the case for complex cells. We demonstrate that as contrast declines both membrane potential and spiking modulation ratios increase, as expected from the recurrent model proposed by Chance et al. (1999).

An alternative explanation for the shift in modulation ratio at low contrast is the variability of contrast response curves across simple cells. Neurons within V1 vary in the contrast at which they saturate, such that for some neurons the changes in contrast may yield large changes in response amplitude whereas for others they may evoke little effect (Van den Bergh et al., 2010). A simple model that includes the variance in the contrast response curves of simple cells which converge onto a complex could only account for input modulation ratio shifts of <0.1, relative to our measures of modulation ratio shifts of >0.45 (data not shown).

We analyzed the responses to drifting gratings and found that there is a shift in the input modulation ratio (V_1_/V_0_) with contrast consistent with a synaptic model (mean V_1_/V_0_ high contrast: 0.62, low contrast: 1.09). While the threshold nonlinearity may play a role in altering the phase sensitivity of neurons (Priebe et al., 2004), there is a clear synaptic component to the shift in phase sensitivity. Also, as expected, when stimulated with high-contrast reversing gratings these cells exhibited various degrees of frequency-doubled responses (Meffin et al., 2015). However, at low contrasts, the same cells showed more modulated, phase sensitive responses to drifting gratings and a tendency to respond to selected spatial phases during stimulation with contrast-reversing gratings.

In summary, for some years now it has been noted that complex cells show increased modulatory responses at low contrasts, suggesting that they are more phase sensitive at low contrasts (Crowder et al., 2007; van Kleef et al., 2010; Henry and Hawken, 2013; Cloherty and Ibbotson, 2015; Meffin et al., 2015). We demonstrate that this is not simply a manifestation of the iceberg phenomenon (Carandini and Ferster, 2000; Mechler and Ringach, 2002; Priebe et al., 2004), but instead a systematic shift in the inputs that cortical neurons receive. This network level change in input modulation with contrast is consistent with a model for the generation of invariant responses in which complex cells emerge steadily through the cortical network through increases in the degree of recurrent inputs that they receive.

## Synthesis

Reviewing Editor: Tatiana Pasternak, University of Rochester Decisions are customarily a result of the Reviewing Editor and the peer reviewers coming together and discussing their recommendations until a consensus is reached. When revisions are invited, a fact-based synthesis statement explaining their decision and outlining what is needed to prepare a revision will be listed below. The following reviewer(s) agreed to reveal their identity: Dario Ringach. Note: If this manuscript was transferred from JNeurosci and a decision was made to accept the manuscript without peer review, a brief statement to this effect will instead be what is listed below.

The revision of the manuscript in response to the previous reviews of the original submission to JN, has now been reviewed by the original reviewers. Serious concerns have been raised by Reviewer 2, which were subsequently discussed with Reviewer 1. Both reviewers noted that the revised manuscript was markedly improved and following the discussion agreed that there are problems remain and those must be addressed before the manuscript will be considered for publication in eNeuro. Below is the list of remaining concerns.

1. The high F1/F0 values may be due to very low F0 that would be indicative of weak neuronal responses. Please address the problem and document that only clearly responsive neurons were included in the analysis.

2. Please address the reservation concerning whether the observed V1/V0 increase is only consistent with just one model. In your revision, allow for the possibility that other models may account for your observations and discuss alternative models that your data rule out.

---------------------------

REVIEWER 1:

Advances in the field:

The manuscript advances the field as it recorded, for the first time, how synaptic input changes as a function of contrast.

Comments to the authors:

The authors have adequately answered my questions and concerns

Additional comments which emerged in the subsequent discussion:

I think it is valuable to document how changes of F1/F0 measured intra-cellularly depend on contrast, and these data are not to be found elsewhere as far as I know. I agree the data, on their own, are consistent with more than one model.

-----------

REVIEWER 2

Advances in the field:

This study investigates the membrane potential dynamics of mouse V1 neurons under different contrasts. The results appeared to be consistent with one computational model, but some important concerns question the overall significance of this paper.

Statistics:

It is critical that the authors only include in their dataset, and the subsequent statistical analysis, cells that were clearly responsive.

Comments to the authors:

The manuscript is certainly improved after the revision to address previous reviews. But I am still not convinced of the significance of the finding (or I just do not fully understand yet).

My main concern in the last review was about the calculation of F1/F0 ratio. The authors have provided details and cited Priebe (2004). The procedure is now clear. But in the Priebe (2004) study of cat V1, only ~2% cells had F1/F0 >2 (Fig. 3a), as one would expect. But in the current paper, 25% cells had F1/F0 >2 (Fig. 4B). In my last review, I noted that the F1/F0 value was as high as 20. Those values can not be determined now as the authors changed the axis range. I have no issues with the new axis range, but those extremely high values, if still exist, are certainly due to low F0, i.e., barely responsive cells. The authors need to make sure that they include in their dataset (and the subsequent statistical analysis) only cells that were clearly responsive.

The heart of my concern, however, is whether the reduced response magnitude, V0, would necessarily give rise to a higher V1/V0 value. As the authors noted in their rebuttal letter, "The point of our paper is that there are signatures of the increase in F1/F0 with decreasing contrast in the membrane potential." If I understand correctly, the higher V1/V0 values under low contrast is the "signature". Is it theoretically possible to have constant V1/V0 under different contrasts? In other words, in order to strengthen their argument, the authors should make a model based on the "iceberg" effect that can simulate the changed F1/F0, but constant V1/V0 under different contrasts.

Furthermore, to generate higher V1/V0 values does not necessarily require "recurrent amplification". The other reviewer raised an excellent point (major concern #2) that the observed result would be consistent with pooling of simple cells with diverse contrast response curves. The authors performed additional simulation and showed the results in the rebuttal letter. It seems that this new simulation actually supports the other reviewer's point. The authors' main argument is that the increased V1/V0 in this new model (0.03-0.14) is smaller than what's observed (0.47). But even in their favored model (Fig. 1C), the increase is quite small (~0.05). Additionally, this new model did not take into account diverse response magnitudes and synaptic strengths as the responses are all normalized. As such, it is possible that a more realistic model could generate a larger increase.

Overall, these considerations question whether the findings in this manuscript would reveal any real insights of visual cortical organization.
